# Supplementary figures and images for: Lek-associated movement of a putative Ebolavirus reservoir, the hammer-headed fruit bat (Hypsignathus monstrosus), in northern Republic of Congo
Source: PLoS One. 2019 Oct 1;14(10):e0223139. doi: 10.1371/journal.pone.0223139 (PMC6772046; doi:10.1371/journal.pone.0223139)

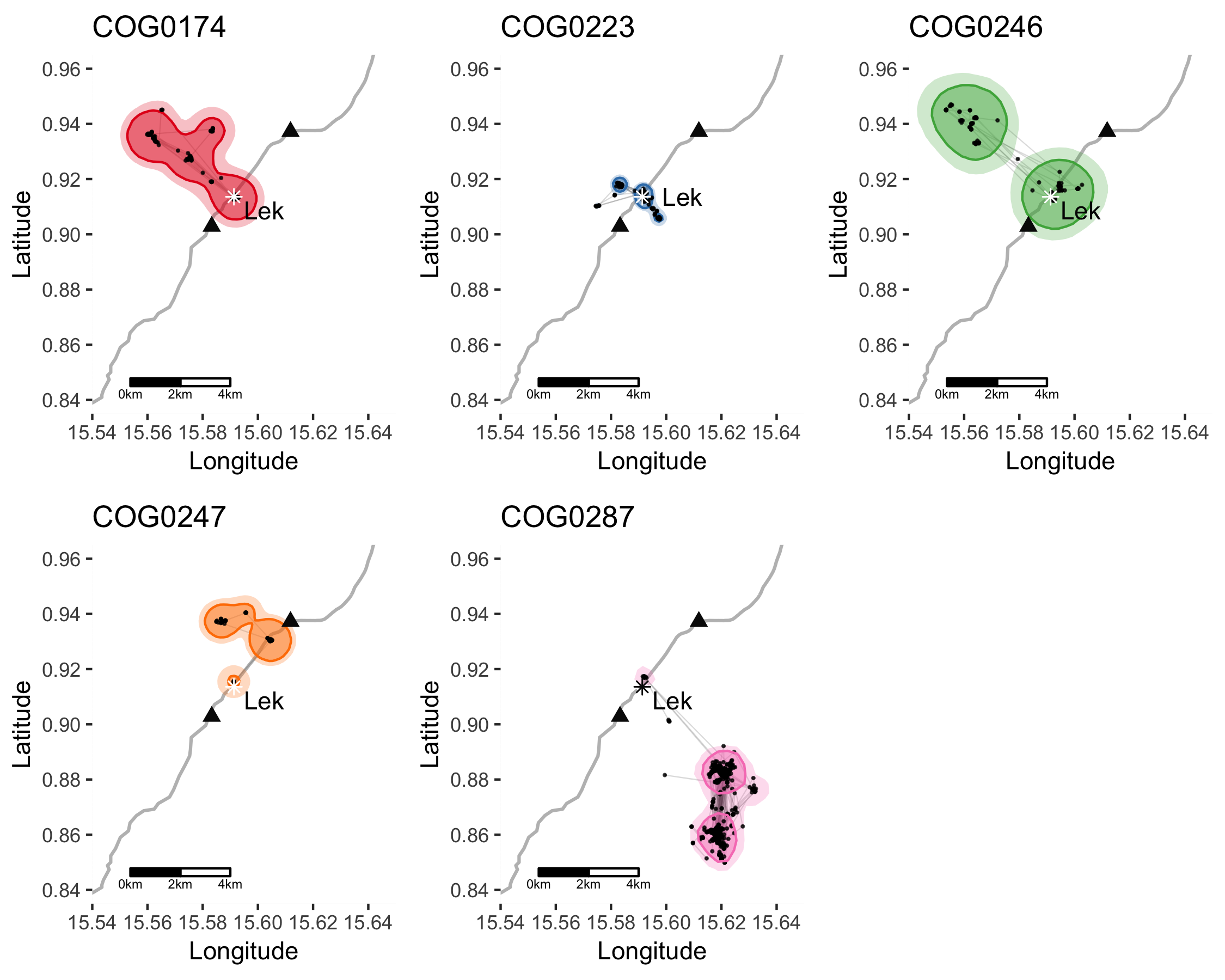

Supplement: S1 Fig — Contours of 80 (darker with outline) and 95 (lighter) percent probability of utilization for each bat. (PNG) [file pone.0223139.s001.png]

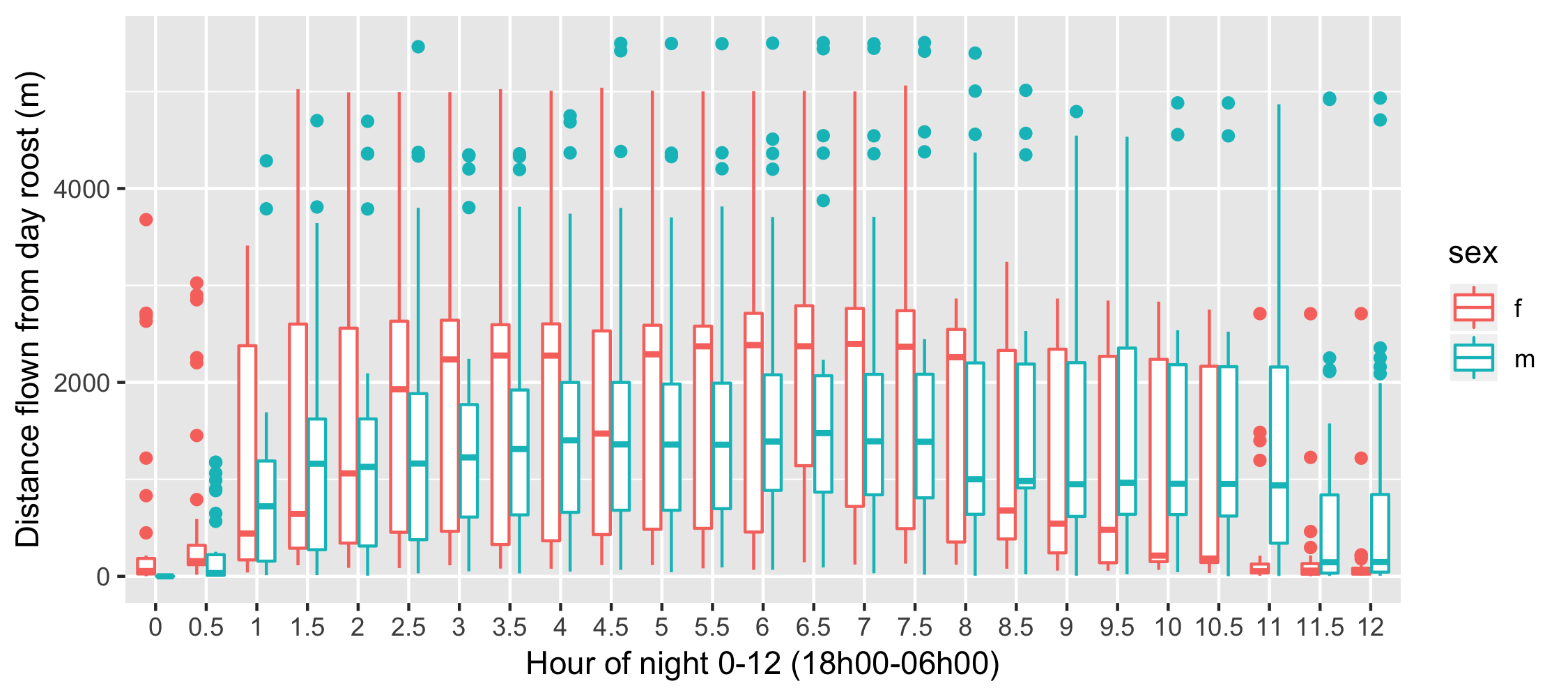

Supplement: S2 Fig — Day roost location is defined as the first GPS location of the evening typically around 18h00 (time 0 in the figure). Displacement is observed over 30 min intervals for all males combined and separately for the female. (PNG) [file pone.0223139.s002.png]

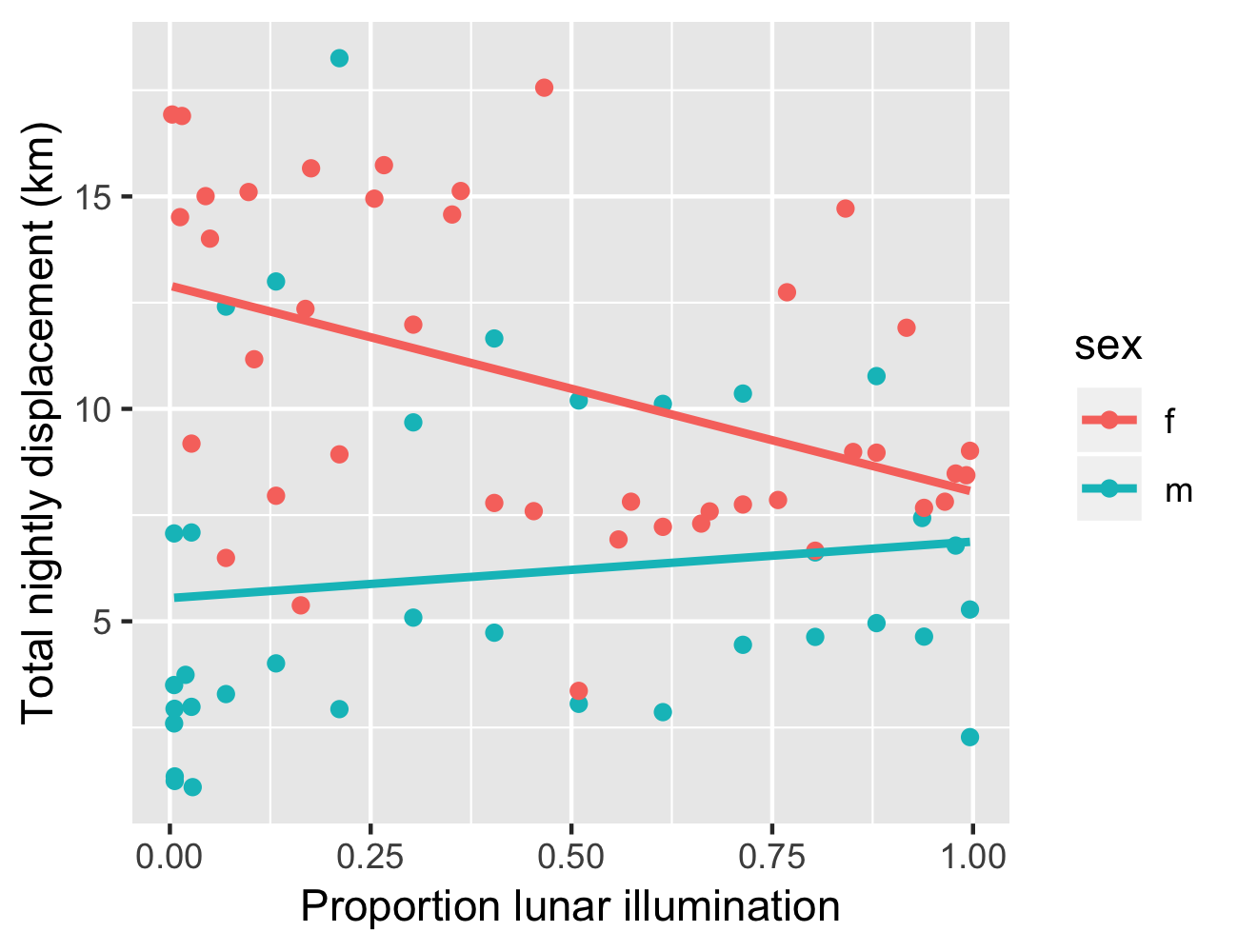

Supplement: S3 Fig — The female’s nightly displacement is negatively correlated with nightly lunar illumination (adjusted R2 = 0.168; intercept = 12898; slope = -4849; p-value = 0.004519). There is no correlation of displacement and daily lunar illumination among the males (adjusted R2 = -0.0148; intercept = 5574; slope = 1329; p-value = 0.480). Data excludes 13 bat nights when less than 10 GPS locations were collected. (PNG) [file pone.0223139.s003.png]
