# Supplementary material for: Lek-associated movement of a putative Ebolavirus reservoir, the hammer-headed fruit bat (Hypsignathus monstrosus), in northern Republic of Congo
Source: PLoS One. 2019 Oct 1;14(10):e0223139. doi: 10.1371/journal.pone.0223139 (PMC6772046; doi:10.1371/journal.pone.0223139)
Supplement: S1 Table — (PDF) [file pone.0223139.s006.pdf]

S1 Table. Utilization distributions (ha) for individual bats calculated using minimum convex polygons (90%) and kernel density estimation (80% and 95%).

Bat ID	Minimum convex polygon (90%) (ha)	Kernal density estimation (80%) (ha)	Kernal density estimation (95%) (ha)
COG0287	615.9	461.5	1010
COG0223	108.5	85.6	185.3
COG0174	547.3	970.6	1621
COG0246	614.5	1293	2414
COG0247	97.07	438.5	829.2
